# Oral Manifestations, Dental Interventions, and Clinical Outcomes in Hospitalized COVID-19 Patients: A Two-Year Cohort Study in São Paulo, Brazil

**DOI:** 10.3390/dj13080362

**Published:** 2025-08-11

**Authors:** Marcelo Ivander Andrade Wanderley, Leticia Rodrigues-Oliveira, Teresa Cristina Dias Cunha Nascimento, Luiz Francisco Cardoso, Thaís Bianca Brandão, Alan Roger Santos-Silva, Ana Carolina Prado-Ribeiro

**Affiliations:** 1 Hospital Sírio-Libanês, São Paulo 01308-050, Brazil; marcelo0andrade@gmail.com (M.I.A.W.); luiz.cardoso@hsl.org.br (L.F.C.);; 2 Oral Diagnosis Department, Piracicaba Dental School, University of Campinas, São Paulo 13414-903, Brazil

**Keywords:** COVID-19, intensive care unit, oral hygiene, dental care, dental needs

## Abstract

**Objectives:** To investigate the demographic, epidemiological, and medical profiles of hospitalized COVID-19 patients who received dental care, and to identify their main oral health needs. **Methods:** This retrospective, descriptive cohort study analyzed medical and dental records of patients hospitalized with COVID-19 at a private tertiary hospital in São Paulo, Brazil, from January 2020 to March 2022. The data collected included demographic variables, comorbidities, length of hospitalization, need for respiratory support, clinical outcomes, dental diagnoses, and procedures performed. **Results:** A total of 129 medical records were reviewed. The sample included 93 males (72%) and 36 females (28%), with a mean age of 72 years. Comorbidities were present in 92% of cases, most frequently a prior COVID-19 infection (59%), diabetes (36%), and depression (31%). The mean hospital stay was 51 days, with a median of 33 days. Most patients (91%) required ICU care; among these, 87% received invasive mechanical ventilation. Dental consultations were most commonly requested for oral assessments (88%), lesions (58%), and opportunistic infections (8%). The most frequent diagnoses were trauma-related lesions from orotracheal intubation (63%), opportunistic infections (45%), and odontogenic or periodontal infections (15%). Primary treatments included oral hygiene procedures (89%), photobiomodulation therapy (67%), and tooth extractions (6%). Patients received an average of eight dental consultations. The overall mortality rate was 26%. **Conclusions:** Older male patients with COVID-19 frequently required intensive dental care during hospitalization. Oral trauma and opportunistic infections were common, highlighting the need for specialized dental management in critically ill populations.

## 1. Introduction

The COVID-19 pandemic imposed an unprecedented burden on public and private healthcare systems worldwide, demanding the mobilization of healthcare professionals, the expansion of hospital infrastructure, increased consumption of medical supplies, and the adoption of strict infection control protocols alongside multidisciplinary treatment strategies for individuals infected with SARS-CoV-2 [1,2,3].

Hospitalized patients are particularly vulnerable to both local and systemic infections, often due to the accumulation of bacterial biofilm on dental surfaces. This disruption of oral microbiome homeostasis facilitates the proliferation and dissemination of opportunistic pathogens [4,5]. The absence of standardized dental care protocols in hospital settings further exacerbates this risk, enabling colonization by microorganisms associated with serious systemic infections. In patients with COVID-19, these complications may be compounded by oral manifestations directly associated with the viral infection, side effects of medical interventions, or the consequences of prolonged hospitalization [6,7].

In this context, dental surgeons have increasingly been recognized as integral members of multidisciplinary hospital care teams, with their role gaining particular relevance during the COVID-19 pandemic—an event that posed unique and significant challenges to hospital-based dental practice. Additionally, the presence of biofilm and poor oral hygiene in the oropharyngeal region has been linked to a range of systemic and oral complications in hospitalized individuals [4].

## 2. Materials and Methods

This retrospective, descriptive cohort study was conducted using demographic and clinical data extracted from the electronic medical records of hospitalized patients diagnosed with COVID-19 who required dental evaluation or treatment. This study was performed at Hospital Sírio-Libanês, a private tertiary care institution in São Paulo, Brazil, covering the period from January 2020 to March 2022. Ethical approval was granted by the Institutional Review Board (or Ethics Committee) of the Instituto de Ensino e Pesquisa do Hospital Sírio-Libanês (CAAE: 61996022.4.0000.5461; approved on 11 April 2023). Informed consent was waived by the Ethics Committee, given the retrospective nature of this study and de-identified data collection. This study was conducted in accordance with the Strengthening the Reporting of Observational Studies in Epidemiology (STROBE) guidelines for observational research [8].

Patients were eligible for inclusion if they were aged 18 years or older, had a confirmed diagnosis of COVID-19, and had undergone a dental evaluation or treatment by the Oral Medicine Team following a formal consultation request. Exclusion criteria were defined as patients who declined oral care, cases in which legal authorization from a designated representative was not obtained, and patients who died before receiving dental evaluation.

Data were extracted from the hospital’s electronic medical record system (Tasy System, HTML5, Philips Clinical Informatics) and subsequently entered into a structured data collection instrument developed on the Research Electronic Data Capture (REDCap) platform (version 14.0.16, Vanderbilt University, Nashville, TN, USA).

The variables collected included the following: sex; ethnicity; age; educational level; comorbidities; medications administered during hospitalization; in-hospital complications; length of hospital stay; unit of hospital admission; need for respiratory support; clinical outcome; reason for dental consultation; total number of dental consultations; dental diagnosis; and treatment performed. For the purposes of this study, “oral trauma” was defined as any ulceration, laceration, or mucosal injury associated with mechanical devices—such as orotracheal tubes or other hospital devices—identified through medical records at the time of evaluation. “Opportunistic infections” included viral (herpetic), fungal (candidiasis), or viral–fungal infections, listed on the basis of typical clinical features such as ulcerated or pseudomembranous plaques and erythematous bases, and, when necessary, corroborated by laboratory or cytopathological confirmation. “Odontogenic infections” referred to infections originating from dental or periodontal sources, evidenced by localized pain, edema, purulence, or radiographic findings described in the medical records. All diagnostic evaluations were performed by specialists in oral medicine and/or hospital dentistry, based on their clinical expertise. No universal classification system was formally applied.

Data collection was carried out between 2022 and 2023 by researcher M.I.A.W. and independently verified by A.C.P.-R. to ensure internal consistency and data reliability.

## 3. Results

Data were obtained from 129 medical records of patients with confirmed COVID-19 who were hospitalized and received dental care during their stay. The study population comprised 93 males (72%) and 36 females (28%), with a mean age of 72 years and a median age of 77 years. The mean length of hospital stay was 29.4 days, with a median of 20 days (Table 1).

Among these patients, 117 (90.7%) required intensive care unit (ICU) support. The mean duration of ICU stay was 46.0 days, with a median of 35.0 days. The most frequently reported pre-existing comorbidities included a prior history of COVID-19 infection (*n* = 76; 59%), diabetes mellitus (*n* = 46; 35.6%), depression (*n* = 40; 31%), and a history of solid organ transplantation (*n* = 35; 27%). Information regarding the comorbidities and respiratory support is summarized in Table 2.

The most commonly administered medications during hospitalization were anticoagulants (*n* = 123; 95%); intravenous corticosteroids (*n* = 100; 77%); antipyretics, analgesics, and antiemetics (*n* = 96; 74%); and antimicrobial agents (*n* = 74; 57%). The most frequent in-hospital complications included myocarditis (*n* = 111; 86%), bleeding events (*n* = 65; 50%), heart failure (*n* = 41; 32%), deep vein thrombosis (DVT, *n* = 27; 21%), and acute kidney injury (AKI, *n* = 34; 26%).

With regard to clinical outcomes, 33 patients (26%) died during hospitalization, while 96 (74%) were discharged. Among those who died, 27 (82%) were male and 6 (18%) were female. Data on the clinical complications and outcomes are presented in Table 3.

Dental care was provided following formal consultation requests to the Oral Medicine Team. The mean number of dental consultations per patient was eight, with a median of five. The most frequent reasons for dental consultation were routine oral assessment (*n* = 114; 88%), evaluation of oral lesions (*n* = 75; 58%), and management of suspected opportunistic infections (*n* = 10; 8%) (Table 4).

The most commonly diagnosed oral conditions included traumatic oral lesions (*n* = 81; 63%), opportunistic infections (*n* = 58; 45%), odontogenic infections (*n* = 20; 15%) and xerostomia or hyposalivation (*n* = 20; 15%). Opportunistic infections were subclassified by etiology: 39 cases (67%) were viral, 12 (21%) were fungal, and 7 (12%) presented concurrent viral and fungal infections. Notably, no cases of bacterial opportunistic infections were identified (Table 4).

The primary dental treatments provided were professional oral hygiene procedures (*n* = 115; 89.1%), photobiomodulation therapy (*n* = 87; 67.4%), and dental extractions (*n* = 8; 6.2%). A summary of dental diagnoses and treatments is presented in Table 4.

Exploratory statistical analyses were conducted to assess possible associations between oral conditions and selected clinical outcomes. The following tables summarize the relationships between the presence of traumatic lesions, opportunistic infections, or odontogenic infections and rates of mortality or length of hospital stay (Table 5 and Table 6).

Patients diagnosed with traumatic oral lesions showed a higher—but not statistically significant—rate of mortality compared to those without such lesions (29.6% vs. 21.3%; *p* = 0.31, chi-square test).

Opportunistic infections were not significantly associated with increased mortality (27.6% vs. 25.2%; *p* = 0.78) but were associated with longer hospital stays (median = 38 days vs. 27 days, *p* = 0.04; Mann–Whitney U test). There was also a significant association between the presence of odontogenic infections and the need for ICU admission (100% vs. 84.6%, *p* = 0.02; chi-square test).

## 4. Discussion

Pandemics and large-scale outbreaks often have sex- and gender-specific impacts, shaped by a complex interplay of biological, social, economic, and cultural factors [9].

According to the Pan American Health Organization, challenges in collecting sex- and age-disaggregated data have hindered a comprehensive understanding of the gender-specific consequences of COVID-19, thereby limiting the development of more equitable and effective public health responses [9]. In the present study, a predominance of male patients (*n* = 93; 72%) was observed among individuals hospitalized due to COVID-19 complications. This finding is consistent with a multicenter study conducted in Brazil by Tem-Caten et al. [10], which reported that men over 60 years exhibited higher levels of inflammatory markers and experienced increased case fatality rates compared to women of similar age.

In terms of ethnic distribution, most participants (*n* = 92; 71%) self-identified as white. According to the Brazilian Institute of Geography and Statistics (IBGE), white individuals in Brazil tend to have higher income levels than Black or mixed-race individuals, a factor that may partially explain their predominance in this study, which was conducted in a private healthcare setting. Nevertheless, broader epidemiological data have demonstrated that Afro-descendant populations in Brazil were disproportionately affected by COVID-19, largely due to structural inequalities such as reduced access to healthcare, overcrowded living environments, and employment in essential but lower-paid occupations [11,12].

Educational background in this cohort also reflected socioeconomic status, with 92 participants (72%) having completed higher education. This is notably higher than the general population, as only 19.2% of Brazilians aged 25 years or older have attained higher education [13]. The elevated educational level in this cohort likely reflects the characteristics of a population receiving care in the private healthcare system. It is also worth noting that the median age in this study was 77 years, a demographic often underrepresented in higher education in Brazil.

A substantial proportion of patients (*n* = 117; 91%) required intensive care support, with a median ICU stay of 35 days, indicating prolonged hospitalization and contributing to a higher risk of complications and poor clinical outcomes. The pharmacological management of these patients included the use of anticoagulants, corticosteroids, and antimicrobial agents such as ceftriaxone, tazocin, and teicoplanin. These therapeutic choices are consistent with the Brazilian Ministry of Health’s national guidelines for hospital management of COVID-19, which align with WHO recommendations [14].

The observed mortality rate of 26% in this cohort is high but comparable to regional statistics. In São Paulo, the COVID-19 mortality rate between March 2020 and December 2023 was reported to be 18.1% among individuals aged 70–79 years and 28.8% for those aged 80–89 years [15]. The advanced age of patients in this study, combined with the presence of multiple comorbidities, likely contributed to adverse outcomes. These findings align with epidemiological data indicating that elderly individuals and those with underlying health conditions are at heightened risk for severe disease and mortality [15].

Dental consultations were most frequently requested for routine oral assessments, followed by the evaluation of trauma-related lesions. These findings are in line with previous research highlighting oral trauma and opportunistic infections as leading causes of dental interventions in hospitalized patients [16]. The most frequently diagnosed oral conditions included traumatic lesions, opportunistic infections, and xerostomia, mirroring findings from prior investigations involving hospitalized COVID-19 patients [7,17]. Among the dental procedures performed, oral hygiene maintenance (*n* = 115; 89%), photobiomodulation therapy (PBMT, *n* = 87; 67%), and dental extractions (*n* = 8; 6%) were the most prevalent. These interventions correspond closely to the diagnoses established. Photobiomodulation therapy (PBMT) was widely used in our cohort, primarily to manage traumatic mucosal lesions and opportunistic infections, as well as to promote local analgesia and accelerate healing in critically ill patients. Although no universal hospital guidelines specifically mandate PBMT for oral lesions, its use has been supported by emerging evidence highlighting its anti-inflammatory, analgesic, and tissue-repair properties. Recent studies have demonstrated favorable outcomes when PBMT is used as an adjunctive therapy for managing oral trauma and ulcerations in ICU patients, including those with COVID-19, contributing to improved patient comfort and faster resolution. Given the high prevalence of intubation-related trauma and mucosal infections in this setting, PBMT represented a feasible, non-invasive, and cost-effective supportive intervention within our hospital’s clinical protocols [16,18,19].

The extended duration of hospitalization, prolonged antimicrobial use, and immunosuppressive regimens required for severe COVID-19 management underscore the critical role of dental professionals within the hospital environment. Their integration into multidisciplinary care teams should go beyond a reactive, on-demand model and move toward proactive, daily engagement. Such an approach can facilitate the early identification and prevention of oral complications—such as trauma, infection, and mucositis—ultimately improving patient outcomes and reducing hospitalization times [18,19,20,21].

Although the primary objective of this study was descriptive, exploratory inferential analyses were conducted to assess potential associations between oral conditions and clinical outcomes. The presence of opportunistic infections was significantly associated with a longer length of hospital stay, suggesting a possible link between mucosal involvement and disease severity. However, no statistically significant associations were observed between traumatic or odontogenic lesions and mortality. These results should be interpreted with caution due to the retrospective design and limited sample size, which may have reduced the statistical power for detecting subtle effects. Nevertheless, the findings underscore the potential relevance of oral health conditions in the overall prognosis of hospitalized patients.

Due to the limited sample size, further stratification of the results by age group or comorbidities was not feasible without compromising statistical power. While such subgroup analyses could offer valuable insights into differential patterns of oral manifestations and outcomes across patient profiles, they were beyond the scope of this exploratory study. Future research with larger multicenter cohorts will be essential to enable robust stratified analyses and to better understand the interactions between demographic, systemic, and oral health variables in hospitalized populations.

Several limitations should be acknowledged. First, this study included only hospitalized COVID-19 patients who received dental care, which may have introduced selection bias by focusing on a specific subgroup already identified as having oral health needs. The absence of a control group (i.e., hospitalized patients without dental consultation) limits direct comparisons regarding the incidence of oral conditions and their potential association with systemic outcomes. Additionally, this study was conducted in a high-income, private tertiary hospital with a patient population predominantly composed of white (71%) and highly educated individuals (74% with undergraduate or graduate degrees), which may restrict the generalizability of the findings to broader or underserved populations. We acknowledge as a methodological limitation the absence of a universally accepted classification system for categorizing oral manifestations. Although the operational definitions adopted in this study—such as “oral trauma,” defined as lesions caused by hospital-related mechanical factors like orotracheal tubes—were consistent with clinical practice and established by oral medicine specialists, the lack of a standardized protocol may limit the comparability of our findings with those of other studies.

Nonetheless, the novelty of this study lies in its comprehensive evaluation of hospitalized COVID-19 patients who received structured dental care within a high-complexity private healthcare system over an extended two-year period. While oral manifestations of COVID-19 have been reported previously, few studies have integrated detailed clinical, demographic, and therapeutic data from dental consultations into a hospital-based cohort with such depth. By documenting the frequency, types, and management of oral conditions—especially trauma related to intubation and opportunistic infections—this study contributes to a growing body of evidence supporting the proactive involvement of dental professionals in critical care settings. Moreover, in the context of a pandemic that strained health systems worldwide, our findings reinforce the relevance of multidisciplinary care models and emphasize the public health importance of including oral health strategies in future hospital preparedness protocols.

Based on these findings, we propose that hospital-based dental teams implement structured preventive measures, especially in ICU environments. Given the high prevalence of intubation-related trauma (63%) and opportunistic infections (45%) in this cohort, daily oral assessments by dental professionals, routine antiseptic oral hygiene protocols (e.g., chlorhexidine rinses), and early intervention for mucosal lesions should be integrated into critical care workflows. Additionally, photobiomodulation therapy may be considered a supportive measure for tissue repair and pain control. These recommendations may help mitigate preventable oral complications, reduce patient discomfort, and contribute to improved systemic outcomes during hospitalization. The integration of such strategies into institutional protocols represents an important step toward safer and more comprehensive care in future pandemic scenarios or similar health crises.

## 5. Conclusions

In conclusion, this study identified that older male patients were the most affected by COVID-19 in the hospital setting and commonly presented with oral trauma and opportunistic infections, necessitating specialized dental care. While the findings contribute to the understanding of dental involvement in COVID-19 care, limitations inherent to the retrospective design must be acknowledged. These include potential inconsistencies in the medical and dental records, and the reduced feasibility of comprehensive oral assessments in critically ill patients. Future prospective studies are warranted to further delineate the role of oral health in systemic disease outcomes and hospital care pathways.

## Figures and Tables

**Table 1:** Sociodemographic aspects of patients included in this study (*n* = 129).

Characteristics	Total No. (%)
Biological sex	
Male	93 (72)
Female	36 (28)
	129 (100.0)
Ethnicity	
White	92 (71)
Brown	13 (10)
Black	5 (4)
Asian	7 (6)
Indigenous	3 (2)
Not defined	3 (2)
Not informed	6 (5)
	129 (100.0)
Level of education	
Uneducated	12 (9)
Middle school	4 (3)
High school	16 (13)
Undergraduate degrees	92 (72)
Graduate degrees	3 (2)
Not informed	2 (1)
	129 (100.0)

Abbreviations: no., total number of patients; %, percentage.

**Table 2:** Medical aspects of patients included in this study (*n* = 129).

Characteristics	Total No. (%)
Unit Hospitalization	
UCI	117 (90.7)
Infirmary	12 (9.3)
Medications administered	
Anticoagulants	123 (95)
Azithromycin	61 (47)
Chloroquine	3 (2)
Hydroxychloroquine	9 (7)
Ivermectin	10 (8)
Intravenous immunoglobulin	10 (8)
Intravenous corticosteroid	100 (77)
Convalescent plasma	7 (5)
Ceftriaxone	74 (57)
Tazocin®	74 (57)
Meropenem	74 (57)
Teicoplanin	65 (50)
Tocilizumab	14 (11)
Remdesivir	12 (9)
Others	96 (74)
No medicine	1 (1)
Types of anticoagulants	
Heparin	60 (46)
Enoxaparin	88 (68)
Other anticoagulants	35 (27)
Observed comorbidities	
Alcoholism	1 (1)
Anxiety	10 (8)
Previous COVID-19	76 (59)
Depression	40 (31)
Diabetes	46 (36)
Dyslipidemia	10 (8)
Systemic arterial hypertension	3 (2)
Hematologic disease	19 (15)
Liver disease	18 (14)
Neurological disease	13 (10)
Oncological disease	28 (22)
Chronic lung disease	1 (1)
Renal failure	9 (7)
Smoking	10 (8)
Previous transplant	35 (27)
Respiratory support	
Ambient air	16 (12)
Oxygen support	113 (88)

Abbreviations: no., total number of patients; %, percentage; UCI, unit care intensive; COVID, coronavirus disease.

**Table 3:** Complications and clinical outcomes during hospitalization for COVID-19 (*n* = 129).

Characteristics			Total No. (%)
Complications			
Bleeding			65 (50)
Pulmonary sepsis			25 (19)
Pulmonary septic shock			2 (1)
Renal replacement therapy			30 (23)
Septic shock—another focus			9 (7)
Disseminated intravascular coagulation			13 (10)
Delirium			1 (1)
Deep vein thrombosis			27 (21)
Pulmonary thromboembolism			3 (2)
Ventricular fibrillation			1 (1)
Ventricular tachycardia			5 (4)
Heart failure			41 (32)
Acute kidney injury			34 (26)
Sepsis—another focus			3 (2)
Severe acute respiratory syndrome			5 (4)
Myocarditis			111 (86)
Pericarditis			4 (3)
Bacterial pneumonia			29 (22)
Polyneuropathy of the critically ill patient			15 (12)
No events			8 (6)
Clinical outcome			
	Male	Female	Total no. (%)
Death	27	6	33 (26%)
Hospital discharge	66	30	96 (74%)
			129 (100.0)

Abbreviations: no., total number of patients; %, percentage.

**Table 4:** Dental aspects of the patients included in this study (*n* = 129).

Characteristics	Total No. (%)
Reason for consultation	
Oral assessment	114 (88)
Traumatic injury	75 (58)
Infectious—focus to be clarified	5 (4)
Opportunistic infections	10 (8)
Xerostomia/hyposalivation	1 (1)
Toothache	4 (3)
Others	10 (8)
Dental diagnosis after oral evaluation	
Oral injury/trauma	81 (63)
Opportunistic infection	58 (45)
Dentoalveolar trauma	8 (6)
Biofilm accumulation	12 (9)
Odontogenic infection	20 (15)
Xerostomia/hyposalivation	20 (15)
Diagnosis of opportunistic infections	
Viral infection	39 (67)
Fungal infection	12 (21)
Viral and fungal infection	7 (12)
Dental procedures and treatments performed	
Soft tissue biopsy	5 (4)
Photobiomodulation	87 (67)
Restorative dentistry	6 (5)
Tooth extraction	8 (6)
Oral hygiene	115 (89)
Periodontal treatment	2 (1)
Installation of intraoral device	8 (6)

Abbreviations: no., total number of patients; %, percentage.

**Table 5:** Association between oral diagnoses and patient mortality (*n* = 129).

Oral Diagnosis	Total (*n*)	Deaths *n* (%)	Survivors *n* (%)	*p*-Value ^1^
Traumatic lesions	81	24 (29.6)	57 (70.4)	0.310
Opportunistic infections	58	16 (27.6)	42 (72.4)	0.780
Odontogenic infections	20	6 (30.0)	14 (70.0)	0.690

^1^ Chi-square test.

**Table 6:** Association between opportunistic infection and length of hospital stay (*n* = 129).

Group	Median Hospital Stay (days)	Interquartile Range	*p*-Value ^2^
With opportunistic infections	38	25–64	0.040
Without odontogenic infections	27	18–51	--

^2^ Mann–Whitney U test.

## Data Availability

The original contributions presented in this study are included in the article. Further inquiries can be directed to the corresponding author.
